# Genetic influences on accelerometer-measured physical activity and sedentary time in children: sex-specific patterns from a Swedish twin study

**DOI:** 10.1038/s41598-026-55559-w

**Published:** 2026-06-10

**Authors:** Björg Helgadóttir, Rui Wang, Ralf Kuja-Halkola, Camilla A. Wiklund, Örjan Ekblom, Maria M. Ekblom

**Affiliations:** 1 Department of Health Sciences, The Swedish School of Sport and Health Sciences, Lidingövägen 1, 114 33 Stockholm, Sweden; 2 Division of Clinical Geriatrics, Department of Neurobiology, Care Sciences and Society, Karolinska Institutet, Stockholm, Sweden; 3 Department of Medical Epidemiology and Biostatistics, Karolinska Institutet, Stockholm, Sweden; 4 Department of Clinical Neuroscience, division of physiotherapy, Karolinska Institutet, Stockholm, Sweden

**Keywords:** Accelerometer, Twin study, Heritability, Environment, Familiar factors, Evolution, Genetics

## Abstract

Physical activity (PA) plays a key role in children’s health, yet it remains unclear why girls are often less active and more sedentary than boys. This study examined genetic and environmental (shared and non-shared) influences on device-measured PA and sedentary time (SED) across girls and boys. This cross-sectional study contained data on zygosity, sex, PA, and SED for 2046 9-year-old twins in Sweden. The accelerometer-measured PA was expressed as the proportion of time in SED, light (LPA), moderate (MPA) or vigorous PA (VPA). Structural equation modelling within a twin framework estimated the heritability of PA and SED, decomposing variance into additive genetic (A), shared environment (C), and non-shared environment (E). Quantitative sex differences were examined by allowing the variance components (A, C, E) to differ across sexes. In the total cohort, genetic contribution was highest in VPA (A_total_: 0.64) but lower in SED, LPA and MPA (A_total_: 0.50–0.55). Boys and girls had differing patterns of genetic contributions, with the largest contributions among girls found for LPA (A_girl_: 0.44; A_boy_: 0.37) while the genetic contribution in VPA was larger in boys (A_girl_: 0.15; A_boy_: 0.57), and the difference was statistically significant for VPA (p = 0.001). While the non-shared environment was similar in all models, the shared environment showed the opposite pattern to the genetic contributions. This twin study shows that genetic influences on PA and SED differ by sex, highlighting the importance of considering how biological mechanisms that drive activity patterns might differ across sex, especially in vigorous physical activity.

**Supplementary Information:**

The online version contains supplementary material available at 10.1038/s41598-026-55559-w.

## Introduction

Physical activity (PA) is associated with the development of motor skills in children^1^. Being physically active as a child has also been associated with multiple health outcomes such as cardiorespiratory fitness, bone health, cardiometabolic health, and depression, as well as having better cognition and school performance^1^. Therefore, low levels of PA among children are a global public health concern^2^. Sex is an important predictor of PA levels, with girls globally having lower PA levels than boys^3^.

Although less conclusive than the evidence on PA, sedentary time has also been linked to several health outcomes in children, such as poorer cardiorespiratory fitness, cardiometabolic health, mental health, and shorter sleep^1^. Accelerometer-measured PA patterns suggest that Swedish 11-year-olds spend 9.7 h a day in sedentary activities, with no sex differences found in this age group^4^. Sedentary time has also been shown to increase by approximately 25 min per year among children of similar age, with larger increases in girls than boys^5^.

The question of why some individuals are less physically active than others is much discussed, and one possible answer is heritability. Heritability is the proportion of the variation of a trait, i.e., PA, that can be attributed to inherited genetics. Twin studies have the ability to disentangle the effects of genes and environment and can also distinguish between the effects of shared environment, i.e., upbringing, and non-shared environment, i.e., experiences unique to each twin. A twin-based study on accelerometer-measured PA in a limited sample of 234 children aged 9–12 years concluded that the shared environment explained 73% of the variance in total PA, with no significant genetic effect, while the children’s enjoyment of PA was largely explained by genetic factors^6^. Similarly, a systematic review of seven studies including between 44 and 624 participants, found that the shared environment explained 60% of the variance of daily-life PA, while genetics contributed 21%^7^.

The knowledge of the heritability of sedentary behaviour in children is lacking. Frequently, studies rely on self- or parental-reported duration of TV viewing or other screen activities. In a sample of 12–18 year olds (*n* = 2847), 75% of the variation in passive sedentary activities was explained by the non-shared environment, while less than 1% was explained by genetics^8^. However, in a study using diary-based data from 659 adolescents and their parents, the results showed that total sedentary time had a genetic component of 27% while specific activities, such as TV viewing only 5%^9^.

Few studies have looked at whether the influence of genetics on PA and sedentary time differs by sex. A higher genetic component was found for boys and men than for girls and women (68% vs. 40%) on sport participation in a sample of 822 adolescents and young adults (12–25 years of age)^10^. Similarly, higher genetic influences were seen for self-reported leisure time PA among boys and men than girls and women (63% vs. 32%) in the same sample^10^. A genotype x sex interaction was seen in a study of sedentary time in adolescents and their parents, with boys having a higher genetic variation than girls^9^.

A better understanding of genetic and environmental influences of PA in children is important for the design of tailored clinical advice, recommendations and other efforts to support child health and development. Although previous studies have explored the influence of genetics and environment on PA, this has seldom been done in a large sample using accelerometers. The strengths of accelerometer measurements include being free of social desirability bias as well as allowing for analyses of different activity intensities. Previous studies have focused on moderate-to-vigorous PA or total PA, while light PA, as well as sedentary time, remain underexplored. Separating moderate and vigorous PA is also of interest, as they have different effects on health^11,12^. Furthermore, twin studies have rarely explored how genetics and environment might contribute differently to PA and especially sedentary time across girls and boys.

### Aims

The aim of this study was to investigate the genetic, shared environmental, and non-shared environmental contributions to light, moderate, and vigorous PA, as well as sedentary time measured with accelerometers. Additionally, the influence of genetics and environment was compared across girls and boys.

## Methods

**Fig. 1:**
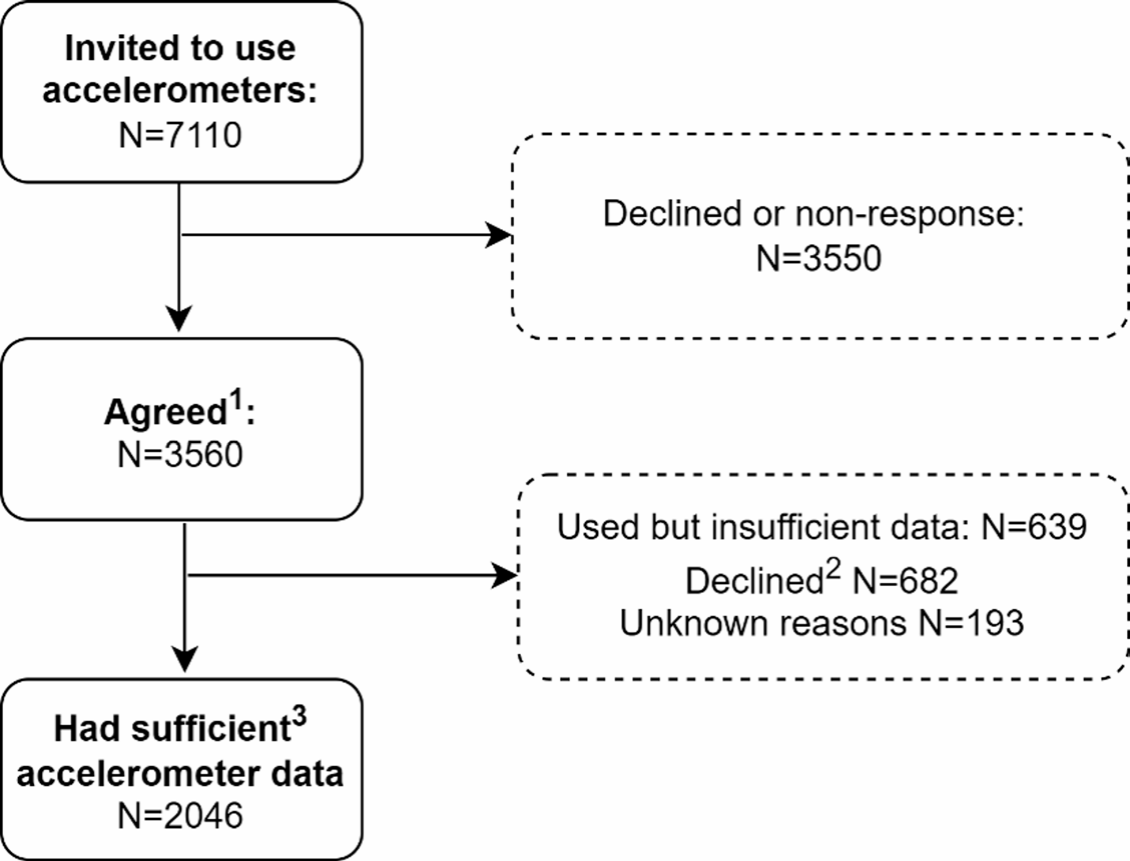
Flow chart of the study participants. ^1^Participant agreed to wear an accelerometer at the end of the questionnaire, ^2^participant later withdrew consent to wear an accelerometer when re-contacted and ^3^sufficient accelerometer data were defined as 4–9 valid days, each with a minimum of 600 min of wear time per day.

The data for this twin-study came from The Child and Adolescent Twin Study in Sweden (CATSS)^13^, which aimed to examine how genetic and environmental factors influence behaviours and health of children and adolescents. Parents of all twins born in Sweden since 1992 have been contacted through the Swedish Twin Registry and asked to answer a questionnaire in connection to their children’s 9th birthday. Since early 2020 the parents were also asked whether the children would be willing to wear an accelerometer, and the current study includes accelerometer data collected between June 2020 and November 2024. In total 7110 individuals were included during this period, of whom 2046 provided sufficient accelerometer data, see description below. See Fig. 1 for more details.

### Accelerometer measurements

Two accelerometers (Actigraph GT3X) were posted to each twin pair and their parents, along with instructions for assigning the correct accelerometer to each twin. The accelerometers were worn for at least seven whole days and returned using pre-paid envelopes. The accelerometer was to be worn on the right hip, except during water activities such as showering. The device should be worn only while the participant is awake; it should be removed in the evening before bedtime and reapplied upon rising in the morning. Parents reported the date and time at which the accelerometer was initially applied and the time at which it was finally removed. These timestamps were used in the analyses to identify and exclude non-wear periods.

The triaxial data for the accelerometers was processed using Actilife (V.6.13.3) into 3-second epochs. Acceleration was measured at 30 Hz. Non-wear time was defined as 30 min of consecutive zeros with no allowance for interruptions. A valid day was defined as 600 min of wear time, which is an accepted limit^14^.Only twin pairs where each twin had between 4 and 9 valid days were included in the analyses as four days is considered the minimum required to reasonably represent a full week^14^. In total 1023 twin pairs fulfilled these criteria.

The accelerometer data was categorised into intensities using the Romanzini cut-offs^15^ (reported for 60-second epochs): Sedentary behaviour (SED 0–720 counts per minute (cpm)), Light PA (LPA 721–3027 cpm), Moderate PA (MPA 3028–4447 cpm) and Vigorous PA (VPA ≥ 4448 cpm). The Romanzini cut-offs have been validated on Actigraph accelerometers in a similar age group^15^. The minutes in each category were averaged across days and converted into percentages of total wear time. Additionally, Moderate-to-Vigorous PA (MVPA ≥ 3028 cpm) and the number of steps were calculated and reported in the supplementary material. Observations of exactly 0 (*n* = 1 in VPA) were changed to 0.000001 as the model does not allow for zeros.

### Other variables

Zygosity was determined through analyses of DNA samples; however, if DNA was not available, it was determined using questionnaire data, i.e., the parents were asked about the similarities between the twins. The zygosity categories were monozygotic (MZ) twin pairs, dizygotic (DZ) same-sex twin pairs and DZ opposite-sex twin pairs. Biological sex was extracted from the Swedish Twin Registry. Parental reports of children’s height and weight were used to calculate Body Mass Index (BMI; kg/m²). Mothers parental education was used as a proxy for socioeconomic status and categorized as elementary school (less than 10 years), upper secondary school (10–12 years) and university or similar (12 years or more). Parents were also asked about the frequency of their children exercising or playing sports during leisure time, with the response options: never, once a week, several times a week or daily.

### Statistical analyses

Descriptive analyses were performed to display zygosity, socioeconomic status, BMI, birthyear, days and minutes of accelerometer wear, and proportions in SED, LPA, MPA, and VPA for the whole group as well as separately for boys and girls. Independent sample t-tests were used to test sex differences for continuous variables, while Chi^2^ tests were used for the categorical variables. Similarly, the differences between participants i.e. those that had sufficient accelerometer data and non-participants i.e. those that did not chose to wear one or had insufficient data accelerometer were also tested.

Pearsons correlations between each twin pair were estimated separately for the proportion of time in each PA intensity. We refer to these correlations as Intraclass correlations (ICC) and they reflect the similarity between twins on traits, i.e., the proportion of time spent in PA intensity. The ICCs were estimated separately for each zygosity and sex combination. If the ICC is higher in MZ twins than in DZ twins, then we can infer that genetic traits play a role in the phenotype (PA intensity), assuming equal environmental contributions to the similarity in the phenotype in both MZ and DZ twins. Based on the assumption that MZ twins share 100% of their segregating genes and DZ twins share on average 50%, we can infer that if the ICC in the DZ twins is more than half of the ICC in the MZ twins, then there is a contribution of the shared environment to the phenotype. Furthermore, if the ICC for MZ twins is less than 1, the remaining proportion is due to non-shared environment contributions to the phenotype. We also calculate the heritability using the so-called Falconer’s formula, i.e., how much of the trait is due to genetic factors compared to environmental factors^16^.

### Twin analyses

To estimate the contributions from additive genetics (A), also known as heritability, shared environment (C), and non-shared environment (E), which includes measurement error, we ran structural equation models, referred to as ACE models. In brief, A refers to additive genetic variance, capturing the proportion of trait similarity attributable to genetic factors. C represents shared environmental variance, comprising environmental influences that contribute to similarity among twins reared together (e.g., socioeconomic status and parental practices). E represents non-shared environmental variance, capturing environmental influences unique to each individual as well as random measurement error, which contribute to differences between twins. These models rely on the assumptions of different genetic contributions among MZ and DZ twins, as described above. Based on this assumption, in the models, the A component was assumed to be perfectly correlated in the MZ twin pairs and the correlation was assumed to be 0.5 in the DZ pairs. The C component was assumed to be perfectly correlated in both types of twins, and the E component was assumed to be uncorrelated in both MZ and DZ twin pairs. Based on these assumptions, expected covariance matrices were modelled to fit the observed covariance matrices from the different twin pairs. In our primary analysis, we initially estimated a constrained ACE model that assumed equal means and variances across sex to maximize statistical power, as is standard practice.

To explore the different genetic contributions across sex we used sex-limitation models that estimate the contribution from the A, C, and E components separately for boys and girls. The differences can be further explored, focusing on qualitative or quantitative differences between boys and girls. Quantitative differences assume that boys and girls are influenced by the same genetic and environmental factors, but to different extents, whereas qualitative differences assume that the genetic or environmental factors themselves differ between boys and girls, e.g., some genes only influence girls^17^. Before running the models, we tested whether means were equal across twin order, zygosity, and sex, and whether variances were equal across twin order, zygosity, and sex, as well as whether variances within sex and zygosity were equal (see Supplementary Table S1). Based on the results of these assumption tests, we concluded that the sex-limitation models with the aim of testing qualitative differences would not be suitable, as the assumptions of the model were violated. However, quantitative sex differences were examined by allowing the variance components (A, C, E) to differ across sexes and only using the same sex pairs. Model fit was evaluated using Akaike Information Criterion (AIC) and Bayesian Information Criterion (BIC). Sensitivity analyses were performed excluding participants with an average daily wear time of 16 h or more, as such excessive wear time may indicate noncompliance with the accelerometer wear instructions. Statistical analyses were performed using R v.4.3.1, using the package OpenMx v 2.21.11 for the twin analyses. For all analyses, α < 0.05 was set as the threshold for statistical significance. All ACE models were unadjusted to capture the total phenotypic variance as observed.

## Results

**Fig. 2:**
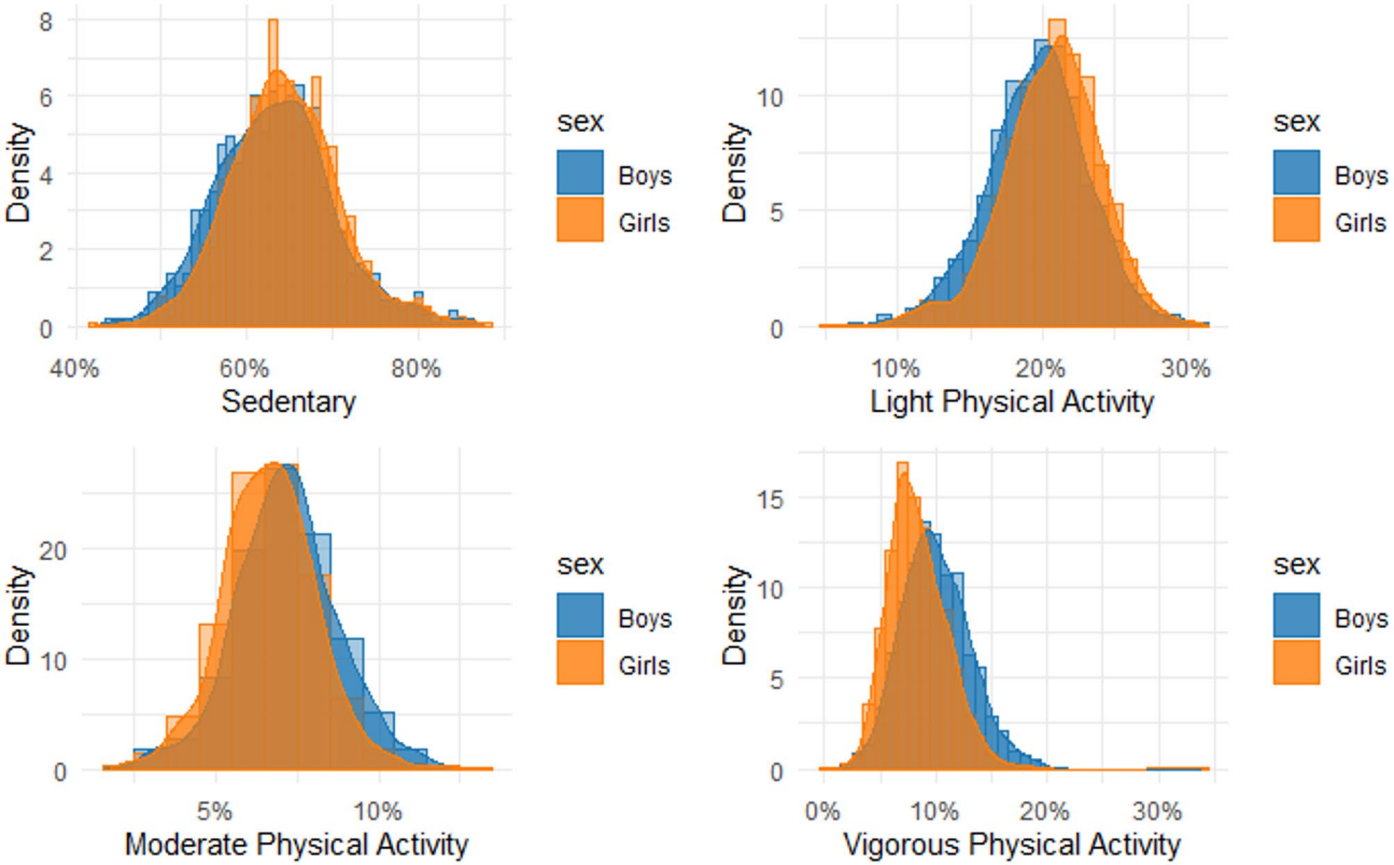
Density graphs for the physical activity variables representing mean percentage of wear time in each intensity for boys and girls.

The sample comprised 2046 twins in 1023 twin-pairs, of whom 48.2% were boys (Table 1). Of the twin pairs, 43.1% were MZ twins, 30.3% were DZ same-sex twins, and 26.1% were DZ opposite-sex twins; the rest were of unknown zygosity. Having seven valid days of accelerometer measurements was most common (47.8%), while 21.7% had six valid days and 16.7% had eight valid days. In the whole group, two-thirds of wear time (awake time) was sedentary time, 20.1% was LPA, 6.9% was MPA, and 9.1% was spent in VPA. Significant sex differences were seen at all intensities. Total wear time did not differ between boys and girls. There were no sex differences between participants and non-participants. However, those with sufficient wear time had a higher socioeconomic status, slightly lower BMI and participated more frequently in sports (see Supplementary Table S2). The distribution of the PA variables can be viewed in Fig. 2.

**Table 1:** Characteristics of the study sample.

	All	Boys	Girls	
	*n* (%)	*n* (%)	*n* (%)	*p*-value
Total	2046 (100.0)	987 (48.2)	1059 (51.8)	
Zygosity				
Monozygotic	882 (43.1)	424 (43.0)	458 (43.2)	0.624
Dizygotic same sex	620 (30.3)	290 (29.4)	330 (31.2)	
Dizygotic opposite sex	534 (26.1)	267 (27.1)	267 (25.2)	
Unknown	10 (0.5)	6 (0.6)	4 (0.4)	
Maternal education				
< 10 years	30 (1.5)	16 (1.7)	14 (1.4)	0.701
10–12 years	528 (26.9)	259 (27.4)	269 (26.3)	
> 12 years	1408 (71.6)	669 (70.9)	739 (72.3)	
Sport or exercise during leisure time				
Never	332 (16.3)	159 (16.2)	173 (16.3)	0.021
Once a week	400 (19.6)	170 (17.3)	230 (21.7)	
Several times a week	1238 (60.6)	611 (62.2)	627 (59.2)	
Daily	72 (3.5)	43 (4.4)	29 (2.7)	
Birth year				
2011	394 (19.3)	200 (20.3)	194 (18.3)	0.026
2012	520 (25.4)	271 (27.5)	249 (23.5)	
2013	472 (23.1)	212 (21.5)	260 (24.6)	
2014	412 (20.1)	178 (18.0)	234 (22.1)	
2015	248 (12.1)	126 (12.8)	122 (11.5)	
Valid accelerometer days				
4 days	66 (3.2)	25 (2.5)	41 (3.9)	0.324
5 days	166 (8.1)	81 (8.2)	85 (8.0)	
6 days	445 (21.7)	209 (21.2)	236 (22.3)	
7 days	978 (47.8)	468 (47.4)	510 (48.2)	
8 days	342 (16.7)	177 (17.9)	165 (15.6)	
9 days	49 (2.4)	27 (2.7)	22 (2.1)	

	Mean (SD)	Mean (SD)	Mean (SD)	
Intensity (mean % of wear time)				
Sedentary time	63.83 (6.55)	63.27 (6.77)	64.35 (6.30)	< 0.001
Light Physical Activity (LPA)	20.13 (3.43)	19.63 (3.43)	20.60 (3.37)	< 0.001
Moderate Physical Activity (MPA)	6.90 (1.53)	7.16 (1.57)	6.66 (1.44)	< 0.001
Vigorous Physical Activity (VPA)	9.14 (2.99)	9.94 (3.03)	8.39 (2.74)	< 0.001
Wear time (minutes per day)	804.2 (90.1)	804.8 (81.3)	803.7 (97.6)	0.768
Body Mass Index	16.4 (2.3)	16.6 (2.3)	16.4 (2.3)	0.076

Table 2 presents the intraclass correlations of the different types of twins in across PA intensities. Correlations were higher in the monozygotic twin pairs, both boys and girls, than in the dizygotic pairs for all intensities. The group with the opposite-sex dizygotic twins had the lowest correlations.

Further, the results showed that the Falconers’ heritability was higher in girls compared to boys across all PA intensities except for VPA. The corresponding results for MVPA and steps can be viewed in Supplementary Table S3.

**Table 2:** Intraclass (Pearson’s) correlations (ICC) and Falconer’s heritability by zygosity and sex.

	ICC SED	ICC LPA	ICC MPA	ICC VPA
Monozygotic boys (*n* = 424)	0.81 (0.75–0.85)	0.77 (0.71–0.82)	0.79 (0.73–0.84)	0.82 (0.77–0.86)
Monozygotic girls (*n* = 458)	0.84 (0.79–0.87)	0.82 (0.77–0.86)	0.77 (0.72–0.82)	0.80 (0.74–0.84)
Dizygotic boys (*n* = 290)	0.62 (0.51–0.71)	0.61 (0.50–0.70)	0.59 (0.47–0.69)	0.55 (0.43–0.66)
Dizygotic girls (*n* = 330)	0.59 (0.48–068)	0.54 (0.42–0.64)	0.53 (0.41–0.63)	0.76 (0.69–0.82)
Dizygotic opposite sex (*n* = 534)	0.49 (0.39–0.57)	0.51 (0.41–0.59)	0.42 (0.32–0.52)	0.37 (0.26–0.47)
Falconers’ heritability boys	0.38	0.33	0.40	0.54
Falconers’ heritability girls	0.49	0.56	0.48	0.07

*SED* Sedentary time, *LPA* Light physical activity, *MPA* Moderate physical activity, *VPA* Vigorous physical activity, all in percentage of wear time.

Table 3 shows the results from the twin analysis of the contribution of the A, C, and E components at each PA intensity, in total and separately for girls and boys. The genetic contribution (A) in the whole group was similar for SED (A_total_:0.50), LPA (A_total_: 0.51) and MPA (A _total_: 0.55) but highest for VPA (A_total_:0.64), while the shared environment (C) showed the opposite pattern. The non-shared environment (E) was similar at all intensities.

The genetic contribution was similar for girls and boys SED (A_girl_: 0.37; A_boy_: 0.39), but the patterns differed by sex in the other intensities. The largest difference was in VPA where the genetic contribution among boys was significantly higher than among girls (A_girl_: 0.15; A_boy_: 0.57; *p* < 0.001). In LPA the girls had a higher contribution of genetics than boys (A_girl_: 0.44; A_boy_: 0.34) while in MPA the opposite was found (A_girl_: 0.31; A_boy_: 0.41). However, these differences were not statistically significant in either LPA or MPA.

The shared environment showed the opposite pattern, i.e., being low for girls on LPA but higher on MPA and vice versa for the boys. For VPA, the contribution of the shared environment was significantly larger for girls compared to boys (C_girl_: 0.66; C_boy_: 0.26; *p* < 0.001). Non-shared environment (E) was similar between boys and girls across all PA intensities, ranging from 0.17 to 0.25. Supplementary Table S4 shows the results from the same analyses for MVPA and steps, both of which had a higher genetic contribution for boys, though only significant for steps.

Model fit criteria (AIC and BIC) indicated that the sex-limitation models consistently showed lower values than the constrained models, suggesting improved relative model fit. Furthermore, sensitivity analyses were conducted including only participants with an average daily wear time of less than 16 h (Supplementary Table S5). The results were very similar to the main analyses for SED, MPA and VPA. However, the genetic contribution for LPA was significantly higher for boys than for girls (A_girl_: 0.54; A_boy_: 0.35).

**Table 3:** Estimated univariate contribution to physical activity patterns from genetics (A), shared environment (C) and non-shared environment (E) with 95% confidence intervals.

	SED	LPA	MPA	VPA
A total	0.50 (0.39–0.61)	0.51 (0.39–0.63)	0.55 (0.42–0.68)	0.64 (0.52–0.75)
C total	0.31 (0.21–0.42)	0.28 (0.17–0.39)	0.23 (0.11–0.35)	0.19 (0.08–0.31)
E total	0.19 (0.16–0.21)	0.21 (0.18–0.24)	0.22 (0.19–0.25)	0.17 (0.15–0.20)
AIC (Akaike Information Criterion)	8655.6	4671.5	2850.1	5176.6
BIC (Bayesian Information Criterion)	−1353.2	−2544.4	−7158.8	−4277.6
A boys	0.39 (0.20–0.58)	0.34 (0.14–0.54)	0.41 (0.21–0.61)	0.57 (0.35–0.78)
A girls	0.37 (0.19–0.54)	0.44 (0.24–0.64)	0.31 (0.10–0.51)	0.15 (0.02–0.28)
C boys	0.42 (0.24–0.60)	0.43 (0.25–0.62)	0.38 (0.19–0.58)	0.26 (0.05–0.47)
C girls	0.45 (0.28–0.63)	0.37 (0.17–0.56)	0.44 (0.24–0.63)	0.66 (0.54–0.78)
E boys	0.19 (0.15–0.23)	0.23 (0.18–0.28)	0.21 (0.16–0.25)	0.17 (0.13–0.21)
E girls	0.18 (0.14–0.22)	0.20 (0.16–0.24)	0.25 (0.20–0.30)	0.19 (0.15–0.23)
*p*-value sex-difference A	0.871	0.496	0.496	< 0.001
*p*-value sex-difference C	0.800	0.628	0.700	< 0.001
*p*-value sex-difference E	0.706	0.349	0.190	0.618
AIC (Akaike Information Criterion)	6275.8	3364.3	1955.7	3814.1
BIC (Bayesian Information Criterion)	−628.6	−1587.0	−4948.7	−3090.2

Total group includes all twinpairs with sufficient data (*n* = 1023) regardless of zygosity. The sex specific groups include only same sex twin pairs: boys *n* = 357, girls *n* = 394.

*SED* Sedentary time, *LPA* Light physical activity, *MPA* Moderate physical activity, *VPA* Vigorous physical activity, all in percentage of wear time, *A* Genetics or heritability, *C* Shared environment, *E* Non-shared environment

## Discussion

This is the first twin-study to use the advantage presented by accelerometers to comprehensively examine light, moderate, and vigorous PA as well as sedentary time in 9-year-old children within a single study. We found that in the total sample, genetic contribution was strongest for VPA. Genetic contributions for VPA were significantly higher in boys than girls, whereas girls had higher, though non-significant, genetic contributions to LPA. Shared environmental effects mirrored these patterns, while non-shared environmental influences remained stable across models.

Previous twin studies in this age group have found a large shared-environmental effect on total PA, with a small or non-existent effect of genetics^6,7^. However, by breaking PA into intensities, a more nuanced pattern emerges. The effects of genetics range from 0.50 to 0.64, being smallest in the sedentary time and LPA, and increasing with higher intensity. The reverse was shown for the shared environment (ranging from 0.19 to 0.31) while the non-shared environment was relatively stable and did not follow a clear pattern. Although non-significant, the effects of the shared environment appeared stronger in sedentary time than for example in VPA. This suggests that these behaviours could potentially be changed using environmental interventions, such as school-based interventions with standing desks to decrease sedentary time^18^. However, it is important to note that estimates from ACE models reflect sources of variance within the sample rather than intervention potential, and stronger shared environmental influences do not necessarily imply greater modifiability.

The sex-specific results showed very similar results for sedentary time, suggesting that changing sedentary behaviours might not depend on sex. Studies on sedentary time and heritability are rare and one previous study concluded that the effects of genetics were higher in boys rather than girls but the results were based on a sample of older children, self-reported data, and the design was family-based i.e., looking at parents and children rather than twins^9^.

Interestingly, the pattern of genetic contributions tended to differ by activity intensity. Girls showed a tendency for higher contributions in LPA and boys for MPA but the only statistically significant difference was observed for VPA, where boys exhibited a notably higher genetic contribution. This does align with a previous study showing that boys and young men have a larger genetic influence for sport participation, which is often focused on higher-intensity PA^10^. There is also some suggestion that there are different sets of genes that influence resting heart rate as well as cardiorespiratory fitness in men and women^19,20^.

This large sample of 9-year-old twins with data collected with accelerometers presents a strong design and methodology to explore the genetic and environmental influences on PA and sedentary time. The data from accelerometers presents a nuanced view of different intensities. Furthermore, self-reported or parent-reported data will inevitably be influenced by recall bias and the tendency to report similarly for both twins, while accelerometers will provide a more objective look at PA patterns. However, one of the limitations of accelerometers is the added burden to participants which could explain why many parents did not agree to use them, limiting generalizability as these participants might have been more interested in PA. Additionally, those with mobility issues might have been less likely to participate. The non-participants had less educated mothers, participated less frequently in organized sports and had slightly higher body mass index, which does limit the generalizability of the results. The Romanzini cut-points for the accelerometer data might have a too low threshold for VPA, as the percentage of time spent in VPA in this sample was high but comparable to other Swedish studies^21^. The cut-points have been validated in a similar age group^15^ and show high agreement to other frequently used cut-offs such as Evenson at least on SED and MVPA^22,23^. Accelerometers do not capture certain activities such as water-based activities and carrying and lifting and hip-worn accelerometers cannot distinguish between sitting and standing. Because the study is cross-sectional, it provides only a snapshot of genetic and environmental influences on physical activity and sedentary time at age 9, limiting inferences about changes in these influences over time. The data did not fulfil the criteria for exploring whether certain genetic or environmental influences were specific to boys or girls.

## Conclusions

This study highlights the importance of sex differences when examining the heritability of sedentary and PA patterns in children. Using accelerometer-measured data from a twin design study, we found that boys show a stronger genetic contribution to VPA than girls. These findings suggest that, at the age of 9, the biological mechanisms driving activity patterns may differ by sex and across activity intensities. Future research should explore the specific genetic and environmental pathways involved, which may inform more tailored interventions to promote physical activity in children. Additionally, following up in a few years will also give information on whether the contributions of genetics and environmental factors change over time and whether these sex differences remain.

## Supplementary Information

Below is the link to the electronic supplementary material.

Supplementary Material 1

## Data Availability

The data that support the findings of this study are available from Swedish Twin Registry but restrictions apply to the availability of these data, so are not publicly available.

## Abbreviations

A: Genetics or heritability
BMI: Body Mass Index
C: Shared environment
cpm: Counts per minute
DZ: Dizygotic
E: Non-shared environment
ICC: Intraclass correlation coefficient
LPA: Light physical activity
MPA: Moderate physical activity
MVPA: Moderate-to-vigorous physical activity
MZ: Monozygotic
PA: Physical activity
SED: Sedentary time
VPA: Vigorous physical activity
