# Supplementary material for: Genetic influences on accelerometer-measured physical activity and sedentary time in children: sex-specific patterns from a Swedish twin study
Source: Sci Rep. 2026 Jun 10;16:17999. doi: 10.1038/s41598-026-55559-w (PMC13254399; doi:10.1038/s41598-026-55559-w)
Supplement: Supplementary file 1 — Supplementary Material 1 [file 41598_2026_55559_MOESM1_ESM.docx]

Table S1: Assumption checks performed before the sex-limitation models

|  | Desired result | SED | LPA | MPA | VPA | MVPA | Steps |
| --- | --- | --- | --- | --- | --- | --- | --- |
| Can means be equated across twin order? | p>0.05 | p=0.306 | p=0.694 | p=0.485 | **p=0.019** | **p=0.041** | p=1.00 |
| Can means be equated across zygosity, within each sex? | p>0.05 | p=0.076 | p=0.276 | p=0.361 | p=0.085 | p=0.092 | **p<0.001** |
| Can variance be equated across twin order? | p>0.05 | p=0.219 | p=0.534 | p=0.533 | P=0.386 | p=0.722 | p=1.00 |
| Can variance be equated across within sex, zygosity? | p>0.05 | p=0.742 | p=0.209 | p=0.329 | p=0.367 | p=0.378 | **P<0.001** |
| Can variance be equated across sex? | p>0.05 | **p=0.013** | p=0.108 | **p=0.042** | **p<0.001** | **p<0.001** | **p<0.001** |
| Can means be equated across sex? | p<0.05 | p=0.019 | p<0.001 | p<0.001 | p<0.001 | p<0.001 | p<0.001 |
| SED: Sedentary time, LPA: Light physical activity, MPA: Moderate physical activity, VPA: Vigorous physical activity, MVPA: Moderate-to-vigorous physical activity in percentage of wear time, all in percentage of wear time. Steps is average number of steps per day. | | | | | | | |

Table S2: Differences between non-participants and participants.

|  | Non-participants | Participants |  |
| --- | --- | --- | --- |
|  | N (%) | N (%) | p-value |
| Sex |  |  |  |
| *Boys* | 2441 (50,4) | 987 (49,6) | 0,105 |
| *Girls* | 2401 (48,2) | 1059 (51,8) |  |
|  |  |  |  |
| Maternal education |  |  |  |
| *<10 years* | 100 (2,4) | 30 (1,5) | <0,001 |
| *10-12 years* | 1325 (31,8) | 528 (26,9) |  |
| *>12 years* | 2740 (65,8) | 1408 (71,6) |  |
|  |  |  |  |
| Sport or exercise during leisure time |  |  |  |
| *Never* | 886 (20,9) | 332 (16,3) | <0,001 |
| *Once a week* | 960 (22,7) | 400 (19,6) |  |
| *Several times a week* | 2262 (53,5) | 1238 (60,6) |  |
| *Daily* | 122 (2,9) | 72 (3,5) |  |
|  |  |  |  |
|  | Mean (SD) | Mean (SD) |  |
| Body Mass Index | 16,6 (2,9) | 16,4 (2,3) | 0,047 |
|  |  |  |  |
| Participants: had sufficient accelerometer data, 4-9 days with at least 600 minutes of wear time. Non-participants: declined to participate or insufficient data.  Note: Twin pairs with one participants and one non-participants were not included in the analyses. | | | |

Table S3: Pearsons correlations and Falconer’s heritability by zygosity and sex.

|  | ICC MVPA | ICC steps |
| --- | --- | --- |
| Monozygotic boys | 0.81 | 0.86 |
| Monozygotic girls | 0.82 | 0.90 |
| Dizygotic boys | 0.65 | 0.63 |
| Dizygotic girls | 0.59 | 0.78 |
| Dizygotic opposite sex | 0.39 | 0.53 |
| Falconers’ heritability boys | 0.32 | 0.47 |
| Falconers’ heritability girls | 0.47 | 0.23 |
| MVPA: Moderate-to-vigorous physical activity in percentage of wear time. Steps is average number of steps per day. | | |

Table S4: Estimated univariate contribution to physical activity patterns from genetics (A), shared environment (C) and non-shared environment (E) with 95% confidence intervals for MVPA and Steps

|  | MVPA | Steps |
| --- | --- | --- |
| A total | 0.62 (0.48-0.76) | 0.57 (0.46-0.69) |
| C total | 0.20 (0.07-0.34) | 0.31 (0.20-0.42) |
| E total | 0.18 (0.15-0.21) | 0.12 (0.10-0.14) |
|  |  |  |
| A male | 0.32 (0.12-0.53) | 0.47 (0.25-0.69) |
| A female | 0.35 (0.14-0.56) | 0.19 (0.05-0.33) |
| C male | 0.49 (0.29-0.68) | 0.33 (0.12-0.54) |
| C female | 0.46 (0.25-0.66) | 0.66 (0.53-0.79) |
| E male | 0.19 (0.14-0.24) | 0.20 (0.15-0.25) |
| E female | 0.19 (0.15-0.24) | 0.15 (0.11-0.19) |
| MVPA: Moderate-to-vigorous physical activity in percentage of wear time. Steps is average number of steps per day. | | |

Table S5: Sensitivity analyses using only those with wear time of less than 16 hours. Estimated univariate contribution to physical activity patterns from genetics (A), shared environment (C) and non-shared environment (E) with 95% confidence intervals.

|  | SED | LPA | MPA | VPA |
| --- | --- | --- | --- | --- |
| A total | 0,51 (0,40-0,62) | 0,58 (0,45-0,70) | 0,54 (0,41-0,66) | 0,62 (0,51-0,74) |
| C total | 0,32 (0,21-0,43) | 0,24 (0,12-0,36) | 0,25 (0,13-0,37) | 0,21 (0,10-0,32) |
| E total | 0,17 (0,14-0,19) | 0,19 (0,16-0,21) | 0,22 (0,18-0,25) | 0,17 (0,14-0,19) |
|  |  |  |  |  |
| A boys | 0,38 (0,20-0,57) | 0,35 (0,16-0,54) | 0,42 (0,22-0,63) | 0,56 (0,35-0,78) |
| A girls | 0,42 (0,25-0,60) | 0,54 (0,33-0,74) | 0,33 (0,12-0,54) | 0,14 (0,01-0,27) |
| C boys | 0,44 (0,26-0,62) | 0,45 (0,26-0,63) | 0,38 (0,18-0,57) | 0,27 (0,06-0,48) |
| C girls | 0,42 (0,24-0,59) | 0,29 (0,09-0,49) | 0,43 (0,23-0,62) | 0,67 (0,55-0,79) |
| E boys | 0,17 (0,13-0,21) | 0,20 (0,16-0,25) | 0,20 (0,15-0,24) | 0,17 (0,13-0,20) |
| E girls | 0,16 (0,12-0,19) | 0,17 (0,14-0,21) | 0,25 (0,19-0,30) | 0,18 (0,14-0,23) |
| *p*-value sex-difference A | 0,751 | 0,036 | 0,516 | 0,001 |
| *p*-value sex-difference C | 0,848 | 0,065 | 0,726 | 0,001 |
| *p*-value sex-difference E | 0,521 | 0,163 | 0,177 | 0,536 |
| SED: Sedentary time, LPA: Light physical activity, MPA: Moderate physical activity, VPA: Vigorous physical activity, all in percentage of wear time. | | | | |
